# The legal determinants of health (in)justice

**DOI:** 10.1093/medlaw/fwac050

**Published:** 2022-12-09

**Authors:** John Coggon, Beth Kamunge-Kpodo

**Affiliations:** Centre for Health, Law, and Society, University of Bristol Law School, UK; School of Law, University of Reading, UK

**Keywords:** Health inequalities, legal determinants, public health ethics, public health law, social justice

## Abstract

The mutual influences of social epidemiology and ideas of justice, each on the other, have been seminal in the development of public health ethics and law over the past two decades, and to the prominence that these fields give to health inequalities and the social—including commercial, political, and legal—determinants of health. General and political recognition of injustices in systematised health inequalities have further increased given the crushingly unequal impacts of the COVID-19 pandemic; including impacts of the legal and policy responses to it. However, despite apparent attention from successive UK governments to injustices concerning avoidable inequalities in health opportunities and outcomes, significant challenges impede the creation of health laws and policy that are both effective and ethically rigorous. This article critically explores these points. It addresses deficiencies in a UK health law landscape where health care contexts and medico-ethical assumptions predominate, to the great exclusion of broader social and governmental influences on health. The article explains how a public health framing better serves analysis, and engages with a framework of justice-oriented questions that must be asked if we are to understand the proper place and roles of law and regulation for the public’s health.

## I. INTRODUCTION: HEALTH INEQUALITIES, INJUSTICE, AND LAW

The links between social justice and health inequalities are empirical as well as critical philosophical matters.^1^ It is well established that significant disparities in health outcomes, and in meaningful (as opposed merely to formal) opportunities to enjoy good health, arise for different groups and communities, and that these are sustained as a result of the configurations and impacts of social structures and institutions.^2^ The direct, causal interactions between health and justice are thus well documented, and captured in the mantra that ‘justice is good for our health’.^3^ Much of contemporary public health ethics, a philosophically-centred field of practical inquiry that became squarely demarcated about two decades ago,^4^ examines the population-level health impacts, and related and surrounding moral obligations, of social and political institutions and organisations.^5^ Within such inquiry, a key area of concern is health inequalities.^6^ Gradations in health opportunities and outcomes relative to socio-economic position have long been recognised, with increasing focus too on additional axes across which relative social (dis)advantage may be measured, such as disability, ethnicity, and gender, and the compounding effect where these intersect.^7^

A forceful representation of the challenges of structural causes of (ill) health, and the problems of relative powerlessness to respond to them simply through enjoining individual responsibility or personal choice, is found in the following table produced by David Gordon and colleagues in the University of Bristol’s Townsend Centre for International Poverty Research.^8^ Their parody of formal, medically -oriented individual advice to address public health challenges is powerful: ‘Don’t be poor. If you are poor, try not to be poor for too long. Don’t live in a deprived area. If you do, move.’ And so on (see Table 1). The framing of the ‘tips’ clearly opens up fundamental questions of shared social, political, and legal responsibility for health.

**Table 1.** Townsend Centre for International Poverty Research, ‘Alternative Top Ten Tips for Health’, available at www.bristol.ac.uk/poverty/healthinequalities.html

	The Chief Medical Officer’s Ten Tips for Better Health	Alternative Tips
1	Don’t smoke. If you can, stop. If you can’t, cut down.	Don’t be poor. If you are poor, try not to be poor for too long.
2	Follow a balanced diet with plenty of fruit and vegetables.	Don’t live in a deprived area. If you do, move.
3	Keep physically active	Don’t be disabled or have a disabled child.
4	Manage stress by, for example, talking things through and making time to relax.	Don’t work in a stressful low-paid manual job.
5	If you drink alcohol, do so in moderation.	Don’t live in damp, low quality housing or be homeless.
6	Cover up in the sun, and protect children from sunburn.	Be able to afford to pay for social activities and annual holidays.
7	Practise safer sex.	Don’t be a lone parent.
8	Take up cancer screening opportunities.	Claim all benefits to which you are entitled.
9	Be safe on the roads: follow the Highway Code.	Be able to afford to own a car.
10	Learn the First Aid ABC: airways, breathing and circulation.	Use education as an opportunity to improve your socio-economic position.
	Source: DoH (1999) Saving Lives: Our Healthier Nation. London: The Stationery Office	Source: Townsend Centre for International Poverty Research, University of Bristol

In this article, our aim is to contribute to critical discourses on the place of law in the context of such debates in the UK, and in particular England. As already indicated,^9^ our ideas about the integration of philosophical ideas of justice with evaluation and proposals for law are informed by works from other jurisdictions.^10^ They also build on a small but growing body of literature within the UK.^11^ Our focus spans across three areas of legal concern:

- the practical impacts (for better and worse) of legal forms of regulation;
- the broader concept of law itself as an overall social phenomenon and source of normative ideas and ideals; and
- the contributions of legal scholarship to practical agendas concerning health inequalities.

The article adds to the growing academic field of public health law,^12^ taking as its central concern the question of how, in the UK, we should approach the ethico-political idea of justice as deployed in agendas to ‘show how the power of law can be used to achieve health with justice’.^13^ Although transdisciplinary approaches to public health law explicitly engage such normative inquiry, they focus primarily on more empirical methods of engagement between law and the social and health sciences.^14^

In what follows, we therefore look to the question of securing the critical underpinnings to claims about how laws, law, and legal scholarship do, can, and should address health inequalities in the UK (in particular with a focus on England). More negatively, this involves a challenge to narrow, medico-ethical and medico-legal framings, and the predominance of paradigms that pretend to ethical neutrality and/or perpetuate impossible demands on individual responsibility through a fixation on civil and political rights to non-interference by the state. More positively, our analysis involves a practical representation of how philosophically-driven approaches from public health may be combined with practical questions in critical legal theory and philosophy to assist engagement and, with the right political will, the achievement of a fairer society. Section II of the article explains why health inequalities are wrongly addressed as a question for medical law and indicates how insights from public health literatures lend perspectives that otherwise could not be drawn. Section III expands on this, identifying in greater and critical detail what it means to take a public health approach. Section IV, with reference to practical questions of justice that have been seen in the context of the COVID-19 pandemic and government responses to it, then introduces a framework that is designed to promote a better marrying of the empirical and critical questions that must be engaged if we are to understand and respond to health inequalities as a problem of law and social justice.

## II. HEALTH INEQUALITIES: A QUESTION FOR MEDICAL LAW?

English medical law, by design, presents conceptual and analytical frameworks that are blind to inequalities. Given the tightness—even the ‘symbiotic’ nature^15^—of medical law’s relationship with ethics, this may seem counter-intuitive. But because of the predominant focus on medical practice and patients’ rights within a National Health Service (NHS), there is a given background assumption of equal access to health care, with entitlement to such access based on the idea of individual clinical need or capacity to benefit, rather (say) than a person’s ability to pay or some form of variable moral desert.^16^ Furthermore, legal adjudication on questions concerning patients’ care does not (formally, at least) give account to competing claims on the necessary resources.^17^ The greater part of the norms of medical law are accordingly focused on individual patients’ rights in the context of individual health care interactions, while across such interactions there is a formal equality that drives analytic assumptions. Patients exist within a system where entitlement to treatment is given based on need, leading in turn to a greater focus on ‘negative’ or civil and political (type) rights aimed at securing protection of individual choice and a defence against unwanted (coercively paternalistic) interference. Such norms are accordingly ill-suited even to raising, less still to addressing, empirical questions of inequality, and related ethical questions of inequity or social injustice.

With that said, it is right to acknowledge that the National Health Service Act 2006 provides an (albeit rather scant) obligation on the Secretary of State for Health and Social Care to ‘*have regard* to the need to reduce inequalities between the people of England with respect to the benefits that they can obtain from the health service’.^18^ There are also medico-legal contexts that give rise to public law considerations and challenges regarding the allocation of resources: these directly shine some light on the systematisation of decisions regarding what care, for what conditions or needs, might be made available or denied (or at least the procedures against which such decisions are made).^19^ And equality protections afforded through the Equality Act 2010 and the Human Rights Act 1998 can and do bear on institutional decision-making, as well as claims brought by individual litigants about what forms of care they may be due. Nevertheless, it remains the case that medical law takes a central focus on hypothetically-isolated clinical interactions between a patient and her carer(s). That patient is presented paradigmatically as a ‘consumer’,^20^ conceptualised as a free decision-maker with an entitlement to be informed, and with rights of choice that are guided by *her* values, beliefs, wishes, and feelings, however eccentric or irrational these may be.^21^

Against that framing, ‘the system’ more widely, and trends regarding different groups or communities within it—including patterns related to health inequalities—are simply irrelevant. ‘Treating patients right’ within English medical law means drawing from a library of contextually-contained rights that would apply at the point of receiving health care.^22^ Little scope exists within medical law’s framings for regard to whether, how, and why there may be material structural distinctions in the ways that members of different groups or communities might exercise those rights, or (unsurprisingly) for non-medical influences on the health that people may, or may not, enjoy. Their primary focus is on what a person is entitled to receive from or refuse from the NHS, with no (direct, anyway) regard for any broader influences on health.

It would, therefore, be odd to imagine that one could or should generalise from principles governing clinical interactions to all other areas of interpersonal and political morality. Nevertheless, the idea of the libertarian person found in the rights-holding medical patient carries a great deal of weight more widely in bioethical thought in the UK, both within and beyond the biomedical sphere.^23^ That person is embodied in the atomised, autonomous individual who may freely declare her own interests, and who enjoys tremendously strong rights with little by way of ethico-legal responsibilities.^24^ Within bioethics, this idea of personhood may be question-begging, wildly overstated, or productively question-raising in its reductive nature.^25^ It certainly has not gone without challenge from wide-ranging scholarly perspectives, including from within medico-ethical and legal literatures.^26^ But insofar as medical ethics draws from works in political philosophy, particular attention, and with it reaffirmation and endorsement of narrowly libertarian ideals and theory, has accordingly been given to (passages of) texts focused on liberal forms of government and their rationales; strikingly, to anti-paternalist tracts within such texts.^27^

What is remarkable in this is not the focus that has been given to patients’ negative rights. Rather, it is the unnecessary and unargued affirmation it gives to wholesale political theories and, for instance, a *general* rejection of paternalism, or a *general* assumption of empowerment being assured through the securing of negative rights. Yet an historical imperative to give greater recognition to *patients’* rights to non-interference need not imply a writ-large endorsement of narrowly libertarian systems of rights, duties, and state powers more generally. Nevertheless, arguments are advanced on the basis, essentially, of medico-ethical norms driving public policy more widely, rather than things working the other way around: norms of and for medicine are given as the starting point for questions regarding health, where the starting point should cover the whole of contexts embraced by politics and political decision-making.^28^ Given what is known about the links between health and social structures, a corrective is needed, rather than an untested assumption within scholarly and practical responses that the paradigms highlighted through medico-legal framings are fit for purpose.

However, just as a great deal of bioethical scholarship may draw too quickly—or without adequate analytical scrutiny—from works in liberal political philosophy, so it is the case that public health agendas, and critical analyses of public health responsibilities in England, are advanced in the shadow of such works.^29^ It is for this reason that public health researchers have observed resort in public health policy to ‘highly agentic’ interventions, and in turn lamented these as a barrier to effective and equitable policy.^30^ Jean Adams and colleagues explain high-agency interventions as follows:

Population [health] interventions … that focus on providing advice, guidance, and encouragement rely heavily on individuals being able and motivated to engage with this advice, guidance and encouragement. These types of interventions have been described as highly “agentic”: recipients must use their personal resources, or “agency,” to benefit.^31^

Policy measures—including the UK Government’s public health plans for England following the onset of the COVID-19 pandemic^32^—are built on express recognition of structurally determined inequalities, while also aiming to come into effect without compromising the rights of the abstract, libertarian person. Significant ethico-political side constraints accordingly stand against (what would be liable to be cast as unacceptable) paternalistic interventions and other representations of institutional interference with personal freedoms.^33^ And this gives rise in turn to arguments that consequent health inequalities will persist. Nutrition and public health experts Bernadette Moore and Charlotte Evans, responding to the government’s obesity strategy, are critical of its emphasis on individual willpower and personal responsibility, without a complementary focus on the need for positive, structured support and resource to provide this: they express concern that the strategy may work best for those who enjoy most structural advantage, and in so doing compound health inequalities.^34^ Such concerns stand alongside a large body of critical public health literature that has challenged the assumptions more widely of public health policy that rests on ideas of consumerism and individual responsibility.

A challenge for health law scholarship that aims to look beyond (hypothetically abstracted) clinical encounters is therefore to revisit the foundational questions of social theory and political philosophy that secure assumptions about what is impermissible, permissible, to be encouraged, or outright mandated. To take seriously concerns about health inequalities, and to be able to frame these as questions of health (in)justice, we should not start from medico-ethical norms. Equally, we need to be prepared to engage with and potentially challenge the libertarian norms more generally that support such policy approaches; including empirical evidence that undermines the concepts (eg concepts of freedom, of the person) on which their normative conclusions are based.^35^ Wherever we ultimately go philosophically, this demands an informed consideration of the practical realities as these relate to the demonstrable impacts of social structures and social institutions, and to the differential consequences of laws and policies.^36^

One of the leading scholars on health justice in the UK and globally, Sridhar Venkatapuram, has made significant contributions given epidemiological research on the socially-determined influences on health that we highlighted in the introduction to this article. Venkatapuram’s position may be seen to raise arguments that run in two directions in such an exercise.^37^ To moral and political philosophers (or lawyers who are influenced by such scholars), he advances a position that says a failure to be able to account for evidence concerning social determinants of health in ethical theorising reflects a fundamental problem with that theorising. In particular, such evidence challenges the rigour and soundness of philosophical assumptions that place store exclusively in individual responsibility. To health scientists—in particular social epidemiologists—Venkatapuram advances a position that draws out expressly how and why their work is not value-free science, but a normatively-oriented endeavour. Following the World Health Organization’s report on the social determinants of health,^38^ he articulates his position in the following terms:

If social factors are identified as determining such significant aspects of human well-being as mortality and morbidity, the moral responsibility for ill health and health inequalities expands beyond the individual to include social institutions and processes.^39^

Venkatapuram’s arguments rest on the matter of demonstrable, empirical fact that individuals alone are not empowered to account for or respond to all of the impacts and influences on their health. The question of *practical* responsibility for health (inequalities) does not therefore move wholesale from asking whether, why, and how individuals can and should be responsible for their own health. But it also calls into the framing—and morally implicates—other actors and institutions. What this means for ultimate moral, political, and legal responsibility is a separate question. There is a difference between identifying regrettable consequences of our social and political systems and in identifying moral failures in political and social responsibility. But crucially, we should not accept philosophical arguments that hold that individual responsibility is sufficient to address responsibility for health (inequalities) where they do so on the basis that individuals alone can determine their health outcomes: to quote again from Gordon and colleagues’ ‘Top Ten Tips for Health’, a person cannot simply choose, for example, not to be poor.^40^ Nevertheless, as we have argued above, predominant medico-legal framings, and their philosophical heritage in libertarian assumptions about individual autonomy and empowerment tend firmly in the direction of saying otherwise, and in so doing foreclose questions that remain to be settled.

To conclude this section, we therefore observe that UK medical ethics and law have developed with predominant assumptions and framings that are ill-suited to addressing health inequalities and associated questions of justice. In the next section of the article, we explain and show how public health approaches are, by contrast, well equipped to problematize questions of health inequalities, and to help identify solutions better to address them through justice-oriented law and policy. As we have argued here, medical law’s contained focus does not allow the development and application of assumptions that may straightforwardly carry into questions of (health) policy writ large. They may even, problematically, be taken without due analysis to affirm and endorse the general soundness and applicability of normative assumptions, for instance, concerning the meaning and scope of individual responsibility for health. In its more doctrinal senses, medical law does not ask, and thus cannot answer, the greater questions concerning health inequalities. And in its more critical and philosophical aspects, it also fails in this regard. Biomedical ethics may include regard to questions of justice; it is, for example, one of the canonical four principles of biomedical ethics.^41^ But to understand the links between inequalities and injustice we need to look beyond medicine, and norms directed at medical and other health care professionals. Public health framings, as we will now explain, better provide the explanatory, conceptual, and critical bases for analyses of health inequalities and laws, law, and arguments made in health law scholarship.

## III. HEALTH INEQUALITIES AND SOCIAL JUSTICE THROUGH A PUBLIC HEALTH PERSPECTIVE

### A. Public health research and practice, and their values-based underpinning

As we will explain, a public health perspective presents various (broadly) unifying conceptual and normative themes. However, to begin to understand how a public health framing may better serve health law analyses, it is important to appreciate that the term ‘public health’ covers multiple, quite distinct ideas, professional identities, areas of policy, scientific approaches, and indeed ideological perspectives.^42^ Research and practice in public health are accordingly not reducible to one role, expertise profile, or speciality. Furthermore, different spectrums may be seen across different characterisations of public health. For example, in public health research we may find more centrally population-focused health sciences, such as epidemiology, being given as the ‘gold standard’. But there are (rightly) challenges to an exclusive or consistently predominant place for them.^43^ Public health is an avowedly multidisciplinary field, engaging researchers from across the health sciences, social sciences, and humanities.^44^ While, within that broad field, disciplines such as law may be viewed as relatively non-central, there is growing recognition of law’s place in public health research^45^ (as well as a longer-standing, if in between times less stated, centrality of understandings of law and legal competences in public health^46^).

Similarly, in practice and policy, there are core identifiable functions and related government powers that are centrally public health in nature; for example, functions in monitoring and responding to outbreaks of infectious diseases under powers provided in the Public Health (Control of Disease Act) 1984. In relation to infectious disease, the potential reach of these powers (which have underscored restrictions regulations in England and Wales during the coronavirus outbreak) are well represented too by the Coronavirus Act 2020. But public health concerns are far more extensive still, and span across government departments and sectors; for instance, education, employment, environment, housing, town-planning, transport—to name just some that we could list—all draw in salient responsibilities regarding the public’s health. Equally, health features as an important consideration when evaluating the rationales for, and proportionality of, public policy; notably as an express consideration given in qualifications to legally protected human rights such as the right to respect for private and family life.^47^ Additionally, forms of regulation that might be deemed public health measures are also affected by and through non-governmental actors, such as supermarkets or community groups.^48^

A single, preclusive characterisation of public health cannot, therefore, be given. It is, though, possible to discern particular features of ideas of public health that sit at the intersection of different understandings of what it means. These in turn circumscribe particular scientific (broadly conceived) approaches and matters of practical concern. Robert Beaglehole and colleagues capture this very effectively with the pithy definition of public health as ‘[c]ollective action for sustained population-wide health improvement’.^49^ This a useful framing for two themes within public health perspectives: the emphasis on health within and across populations; and the emphasis on interventions that are effected through collective measures (or put another way, effected through modes of social coordination including law and regulation).^50^

As explained in Geoffrey Rose’s seminal paper ‘Sick Individuals and Sick Populations’, public health sciences look to what we learn when we make observations about health by studying populations.^51^ Doing so gives rise (amongst other things) to distinct sorts of inferences about causes of ill health: looking at the distinct incidence of (say) cardiac disease in two populations allows for the consideration of causal factors that are not discernible or demonstrable when considering an individual case, or reducible to issues that are within the control of any given individual. What might be labelled ‘the cause’ of an individual’s heart attack (eg a sudden physical exertion) is quite distinct from what might be given as the causes of higher incidence of poor cardiac health in one population compared with another (eg considerations around diet, exercise, genetics, and so on).

And just as observations and understanding may differ when we look at a population level, so may our interventions when ‘treatment’ is of the ‘population as a patient’.^52^ As a framing for policy approaches, population-level interventions incorporate measures that are designed to reduce the incidence of disease by targeting groups. This includes targeting low(er)-risk populations (for example by decreasing general levels of salt consumption or recommending use of statins), rather than simply responding to high-risk individuals with a remedial intervention after ill-health materialises (say by fitting a stent after a person has had a heart attack). As a matter of professional and social ethics, such policy approaches give rise to questions of political morality and social justice: for instance concerning paternalism, the (re)distribution of resources, and the relative inclusion or exclusion of different groups and communities in public decision-making. They also give rise to what Rose labelled the ‘prevention paradox’: the practical and ethico-political challenge of subjecting people to regulation (eg to reduce salt consumption) that will show health improvements at a population level, while placing regulatory burdens on people whose individual risk of harm is relatively low and for whom any individual health benefit may never be demonstrable.^53^

In its core senses, public health is therefore unavoidably political, intertwining concerns for scientific rigour with ideas about ethical values and social equity. This has direct implications for the roles and remits of public institutions and the communities that they serve. In the phrase of Richard Horton, editor of *The Lancet*, ‘public health is the science of social justice’.^54^ It is important to emphasise this, as the point can be missed, or even obfuscated. Responding to the UK government’s early responses to the COVID-19 pandemic, ethics experts pushed back against the idea that public health decision-making could simply be about ‘following the science’.^55^ Such a point is long and well understood by members of the public health community, whose scientific and practical roles are—whether they like it or not—represented as placing them in ranging positions of social and political activism.^56^ When she was England’s Chief Medical Officer, Sally Davies, writing with colleagues, outlined how historical developments in public health sciences reflected distinct policy agendas and regulatory approaches in support of values-based goals, as well as advocating for the direction in which governance for the public’s health should now move.^57^ The significant question to consider thus is not *whether* values are at play, but *which* moral values should inform the core of public health and from there come to direct policy and practice? Section II of this article has shown how and why medico-legal framings are ill equipped for this task. Exploring the question from a cross-societal, cross-sector, population perspective allows us to see how studies in public health ethics and law may much better engage with questions of inequality and injustice.

### B. The ‘Moral Mandates’ of public health and responsibility for effective responses to avoidable Ill health

As Kathryn MacKay argues, it is sometimes the case that public health ethics is represented as espousing a blunt and monistic, maximising moral system (often presented as utilitarianism).^58^ On this take, the concern would simply be about achieving the highest possible aggregate levels of health within a public, with regard neither to moral concerns for individual rights, nor for the distribution of how and by whom health is enjoyed across that public. Such a representation, however, if given to capture all of public health ethics, is caricature. It misses what MacKay refers to as ‘the equity view’.^59^

In line with MacKay’s observation and its underlying concerns, the ethics paper that supports the Public Health Skills and Knowledge Framework for the UK’s public health workforce explains how public health research and practice are widely recognised as resting on a mission to address two particular sources of moral concern.^60^ There is indeed a more maximising ethic to protect and promote health. This comes through general preventive measures to stop or limit the incidence of disease, illness, and injury, such as assuring clean environments (including eg workplaces), access to clean water and safe and nutritious food, providing interventions such as vaccine programmes, regulating for road safety, and so on. And it comes through general measures to sustain and improve good health (often with a focus too on positive states of well-being), such as by attending to mental good health as a core aspect of education and employment, or ensuring that people have meaningful access to opportunities for recreation.

However, that maximising ethic is complemented—and sometimes constrained—by a distinct, egalitarian ethic to prioritise addressing avoidable, systematised health inequalities.^61^ As explained in the introduction to this article, historically, public health research and practice in the UK have been especially concerned with unequal enjoyment of health (opportunities) measured against relative points of socio-economic (dis)advantage; but with increasing recognition of further axes against which health inequalities might be identified, such as ethnicity and gender, through intersectional approaches.^62^ We accordingly also find that ideas of *equity* or *fairness*—of *distributive justice*—are foundational within the ethics ‘of’ public health.^63^ These temper any maximising imperative with justice-based imperatives that provide proportionately greater attention to groups or communities who face particular, socially-generated disadvantage. As Michael Marmot and colleagues write in their seminal report on English public health policy, *Fair Society, Healthy Lives*:

To reduce the steepness of the social gradient in health [i.e. to provide greater health equality across distinct points of social position], actions must be universal, but with a scale and intensity that is proportionate to the level of disadvantage. We call this proportionate universalism. Greater intensity of action is likely to be needed for those with greater social and economic disadvantage, but focusing solely on the most disadvantaged will not reduce the health gradient, and will only tackle a small part of the problem.^64^

These ideas capture the ‘moral mandates’ of public health.^65^ It would be simplistic to suggest that there is a single, universally-held moral outlook or social agenda shared by everyone who takes a public health approach.^66^ But in summary, we find an overwhelmingly predominant commitment to the following two imperatives:

- First, health opportunities and outcomes are to be maximised both through proportionate preventive measures to defend against disease, illness, and injury, and through proportionate health-promotion interventions to sustain and enhance general levels of health (and on many counts well-being); and
- Secondly, systematised, avoidable, and unfair inequalities in health (opportunities) must be addressed: social architecture that supports or creates differential enjoyment of health rests on poor foundations, and priority should thus be given to protecting and promoting the health of groups and communities who face greater disadvantage.

In relation to both of these moral mandates, and recognising that they may, at times, stand in tension with one another, insights from a public health perspective take us through considerations of what instances of poor health should be of concern, and where responsibility for addressing them should lie.^67^ If health is either to be better protected through guards against disease, illness, and injury, to be promoted through attention to assuring positive well-being, or to be more equally enjoyed, we need to understand this practically and ethically—as explained and argued so forcefully by Venkatapuram^68^—by reference to the question of whose actions or inactions give rise to responsibility for health (inequalities).

Effectively assuring conditions in which people can enjoy good health, and addressing avoidable inequalities in health, in public health parlance, includes a great concern for looking to (often complex networks of) ‘upstream causes’^69^ or ‘causes of causes’.^70^ Rather than focus just on individual-level, responsive, remedial health care, we must look much more widely, and aim to anticipate threats to health by reference to multiple influences. In its agenda-setting report *Improving the Health of the Public by 2040*,^71^ the Academy of Medical Sciences talks of the importance of focusing much more on measures directed to prevention of disease and harms to well-being. It focuses in particular on recalibrating agendas to account for ‘primary prevention’, explaining preventive interventions as follows:

In terms of health, prevention involves a range of interventions aimed at reducing risks or threats to health. Primary prevention aims to prevent disease or injury before it occurs, for example by immunisation, health education and preventing exposure to hazards. Secondary prevention aims to reduce the impact of a disease or injury which has already occurred, for example by detecting, diagnosing and treating as soon as possible as well as taking steps to prevent reoccurrence. Regular screening programs, such as mammograms for detecting breast cancer, are an example. Tertiary prevention aims to reduce the impact of a disease or illness which is ongoing and has long-term effects, by helping people to manage often complex health problems and injuries to maximise their quality of life and life expectancy. Rehabilitation and support programs are forms of tertiary prevention.^72^

Given the practical understandings that public health research gives us of causes of avoidable ill health and health inequalities, why do they persist so forcefully? Why do we not have better systems of primary prevention? The ideas of assuring better and fairer health opportunities and outcomes are constructed around scientific evidence bases that explain both how and why we find incidences of poorer health, and what practical measures would address these.^73^ Successive UK governments have long been aware of the material fact of health inequalities, of their worsening over time, and of their causes being broad-reaching rather than a problem that may be fixed through the NHS.^74^ In the next section of the article, we look to the critical implications of these questions given the renewed recognition of health inequalities, and their status as a question of social justice, in light of the COVID-19 pandemic.

## IV. COVID-19 AND A RENEWED RECOGNITION OF HEALTH INEQUALITIES AS A PROBLEM OF SOCIAL JUSTICE

### A. COVID-19, health inequalities, and the structural determinants of social (in)justice

As explained in Section III of this article, advocacy to respond to health inequalities is a longstanding concern in public health and public health ethics. But within public discourses consequent to the onset and impact of the COVID-19 pandemic, there has been a renewed recognition of health inequalities as a problem of social justice. While everyone has been affected by COVID-19 and measures put in place against it (eg general restrictions regulations), the harms and burdens have not been equally spread out within societies or dissociated from pre-existing structural determinants of unequal enjoyment of health (and other markers of social injustice). Rather, they have fallen along racialised,^75^ gendered,^76^ ableist,^77^ and other lines. In other words, within the course of the pandemic, people in the UK may have faced the same storm, but from within very different boats.^78^ COVID-19 has magnified pre-existing health and other inequalities, while also creating new ones. For example, it has shown how pre-existing income and wealth inequalities make some ethnic minority communities in the UK more vulnerable to living in over-crowded accommodation, which directly increases the risks of transmission and infection of the disease.^79^ Additionally, the pandemic has shone a spotlight on pre-existing digital exclusions and divides,^80^ meaning that more vulnerable children were ill equipped with the technology they needed for home schooling prior to Government intervention.^81^ Furthermore, questions of the deeply and unfairly segmented nature of the UK labour market^82^ have been revived during COVID-19, as it became clear that poorer people and especially those from ethnic minority backgrounds were more likely to work in occupations that were more public facing, and/or could not be done from home, and/or were those in which ‘social distancing’ precautions were difficult to observe.^83^ While from early on in the pandemic it appeared that men have been more at risk of death from COVID-19,^84^ women of different races and social classes have been at higher risk of the incapacitating effects of long covid,^85^ while at the same time having an increase in care responsibilities particularly during the national and regional lockdowns.^86^

Because health inequalities are avoidable through changes to social norms, structures, and institutions, their very existence makes them a question of social justice.^87^ In Section II of this article, drawing on Venkatapuram’s argument, we explained how critical and practical discourses on social determinants of health injustice require combined engagement from health sciences and critical fields such as political philosophy. Section III has explained how public health perspectives shed particular light, and how critical framings have emerged from within public health in relation to protecting and promoting health and reducing health inequalities. Here, we wish to complement that critical framing with reference to terms provided from critical theory regarding ethics, politics, and law. These, we argue, both inform discourses from a public health perspective, and help to orient where UK health law scholarship should direct itself when scrutinising health inequalities.^88^

If we engage these literatures, rather than consider questions of health inequalities with blunt and (essentially) exclusive reference, for instance, to liberal conceptions of autonomy and the need for defences against state regulations and professional hegemonies, we see causation within a ‘matrix of domination’,^89^ whereby several intersecting systems of oppression collude^90^ to produce glaring disparities based on race, gender, class, geographic region, dis/ability, sexual orientation and so on. For example, there is clear evidence of how racism, sexism and classism collude to produce unequal access to quality education,^91^ ability to secure reliable employment that pays liveable wages,^92^ and the ability to live in quality housing.^93^ These all exacerbate health inequalities.^94^ Intersecting inequalities make it harder or impossible for people in low-paying, precarious, and/or hazardous work to switch employment. Pre-existing inequalities also make poorer people more vulnerable to shocks in the system; for example, to mass unemployment during times of economic difficulty, such as during the first COVID-19 lockdown when the UK economy was declared to be in ‘severe recession’.^95^ Insufficient income further contributes to the inability to afford nutritious food in adequate amounts, leaving people even more vulnerable to having to resort to emergency provisioning such as the use of food banks.^96^

It is at such points that insufficient explanations and framings for the existence of health inequalities become more stark, and the substantiation about claims that they are unfair may crystalise. Through that process, we also may identify what it means to address them head on if law (and policy) are to respond to questions of health injustice, rather than be complicit in their causal structures. Some explanations for inequalities during the COVID-19 pandemic have been marked by wilful ignorance of systemic factors. For example, the UK Government, through a report published by its Commission on Race and Ethnic Disparities on 21 March 2021, dismissed state-sanctioned racism as a contributor to health inequalities.^97^ This is despite evidence to the contrary, including evidence by Public Health England on the racialised nature of COVID-19 harms,^98^ and evidence of the effects of systemic racism in the NHS.^99^ Other explanations are ahistorical, failing to consider how the effects of the COVID-19 pandemic were made even worse by a decade of austerity. For example, austerity cuts directly impacted on the lack of preparedness of the NHS,^100^ and insufficient stock-piles of Personal Protective Equipment (PPE). The absence of PPE, especially during the first national lockdown in 2020, directly contributed to avoidable deaths particularly of NHS workers, many of whom were from ethnic minority communities.^101^

In conclusion, while COVID-19 has brought health and other inequalities into sharp relief, it would be mistaken to assume that if there had not been a pandemic then the inequalities would not have existed, and/or that health inequalities would disappear after the pandemic ‘ends’. The circumstances pre-existing the COVID-19 pandemic, such as harsh austerity measures for a decade and deep welfare cuts, directly created the context for the inequities and many of the avoidable harms, that have resulted from the coronavirus and responses to it. For example, the use of food banks, including by people in full time employment,^102^ was already increasing at an alarming rate prior to the pandemic.^103^ As has been argued elsewhere,^104^ ‘normal led to this’.^105^ Therefore, even though the failure of pandemic preparedness in terms of resources (eg stockpiling personal protective equipment) and local administration was already significant, there would still have been (and emphatically there were) systemic harms reflected in health inequalities. We should therefore be wary of narratives that suggest an ‘inevitability’ of the harms and unequal impacts that have resulted from COVID-19 pathogens and responses. Health inequalities were not an aberration of the pandemic, but were baked into the system. COVID-19 has reminded us that ‘the system isn’t broken, it was built this way’.^106^ And the way the system is built reflects philosophical commitments and assumptions. As indicated in the previous two sections of this article, while predominant framings from and in medical—or even health care—law cannot satisfactorily address these, the combined theoretical and practical resources of public health can do. The literatures on public health ethics and law have sought to bring these to bear, with various outstanding contributions (more from other jurisdictions,^107^ but including within the UK^108^).

In the final section of this article before the conclusion, we take the practical points regarding inequalities as exemplified against narratives that have come to the fore during the COVID-19 pandemic, and indicate how approaching these through a public health perspective and as a matter of social justice can work. This is not with a view to being comprehensive in representation of ideas of justice, or to advancing or defending a single or preclusive idea of justice; rather, it is to show the sorts of prior questions that must be asked, and to indicate the critical scope that any response to them must have.

### B. Health inequalities and legal scholarship: towards fuller understandings of injustice and health

To begin a critical re-evaluation of law’s place in relation to health inequalities, we would promote research agendas that take an adapted version of a framework of four questions concerning social injustices, health inequalities, and COVID-19, which was developed by one of this paper's authors as part of the UK Pandemic Ethics Accelerator project.^109^ The questions are inter-connected and whatever answers may be given to them are part of the same dialogue: we need to anchor ideas of moral responsibility to values that matter, but also to actors whose responsibility can meaningfully be said—and shown—to be at play: individuals understood within their lived realities; and other actors and agencies, including of course public ones. We advance these questions here noting that they neither come from nor invite a singular critical, ethical, or political theoretical framing. They rather invite insights from a plurality of literatures, and the identification of and engagement with a plurality of perspectives. The framework from which they are drawn explains more fully the general importance of these questions, while also advancing its own, contextualised, critical narrative against the UK’s public health and political landscapes.

First, *whose care (broadly conceived) is constructed as the type of care that can consistently be left waiting?* This question does not prompt us to challenge the idea of prioritisation questions *per se*. In any resource-limited system, there will always be the need to prioritise due to finite resources (including but not limited to financial resources). The question instead prompts us to consider systemic neglect and care-lessness aimed at communities. Scholarship on health justice needs to pay attention to the serial disregard for particular groups and communities.^110^

Secondly, *who does not get to breathe?* The summer of 2020 was filled with chants of ‘I can’t breathe’ in protests that erupted after the killing of George Floyd through police brutality.^111^ One outcome of health injustices can be loss of life. At the same time, health injustices can also cause a slow death that comes from laws and policies that are oppressive and do not leave any room for respite. For example, laws and policies can, in essence, prioritise the interests of commercial actors; phenomena explored in critical public health literatures on the commercial determinants of (ill) health.^112^ Or, as one more example, immigration laws and policies can be—and are—purposely aimed at creating ‘hostile environments’ to particular populations such as asylum-seekers, in ways that negatively impact on physical, mental and emotional health.^113^ This second question aims at helping us consider whose comfort and well-being the law centres, and who is left frazzled and grasping for breath.

Thirdly, *whose voices are not being heard?* This is a basic as well as a central question in any policy evaluation. At the heart of justice is listening to voices; particularly those that are more likely to be erased.^114^ Failure to listen is itself a form of violence: epistemic violence.^115^ This, again, is based on and fuels other injustices such as testimonial injustice,^116^ whereby a speaker’s credibility is reduced because of their race, gender, class, disability, sexuality or other social locations. Dialogues and listening help us to make more sense of how health-influencing goods become unequally distributed.

Fourthly, *what outcomes are truly (not) inevitable?* This question helps us to scrutinise explanations as to why things are the way they are. Sometimes health inequalities are seen as inevitable because, for example, their causes are framed as a manifestation of a culture of a particular population.^117^ Or inevitability can be based on false biological essentialism (‘it is just the way women are’ for example).^118^ Such faulty framing either places responsibility in the wrong place, or denies that those in power can or should take responsibility. Seemingly ‘inevitable’ consequences of societal and political configurations can be challenged if we take seriously and scrutinise the related empirical and ethical ideas of responsibilities (causal and moral) being diffused across individuals, communities, organisations, and governments. A *status quo* bias, or a hearkening to the priority of what has been ‘normal’, requires as strong a defence in terms of justice as any challenges to it.

## V. CONCLUSIONS

Health inequalities indicate problems of social injustice. Specifically (and tautologically), they are problematic insofar as they are unfair and avoidable through means that themselves are morally-mandated. That point bears emphasis because, as we have spelled out, these properties of unfairness and avoidability require us to bite political bullets. Wherever we sit in political philosophical terms—whether in a more libertarian or more collectivist or communitarian camp^119^—it is incumbent positively to provide and defend a particular account of, or approach to, understanding justice; and in so doing, to be able to defend what this means in terms of outcomes such as health inequalities. This will allow us to evaluate law’s place within structural determinants of health; how laws are or may be complicit in creating and sustaining, or how they do and may serve to guard against, health injustices.

The public health ethics literatures provide excellent examples of works that take on this task. And recognising the growing legal scholarship that engages and contributes to these,^120^ we would argue that public health law literatures have a more pronounced role to play here in integrating themselves with the express engagement of philosophical ideas of social justice. The timeliness of such a direction in legal scholarship was marked by the publication of the *Lancet*-O’Neill report on the legal determinants of health.^121^ And its necessity is undeniable following the central—and highly contested—role of law and policy following the onset of COVID-19 to all manner of questions concerning influences on the public’s health. The ideas of responsibility for protecting and promoting health *with justice* have divided people in the UK. The pandemic has seen the implementation of sweeping and draconian statutory measures, both under the Coronavirus Act 2020 and through secondary legislation made under the Public Health (Control of Disease) Act 1984, with consequent (and ongoing) reviews by Parliament,^122^ ranging organisations,^123^ and even the courts facing questions such as challenges against the lawfulness and proportionality of restrictions regulations.^124^ From within and beyond the pandemic, across sectors, we may see three vital functions—and challenges—for law and laws as these impact the public’s health and health inequalities, and the roles and forms of legal scholarship in addressing them.

First, we may look at law as a key structural aspect within the social determinants of health: laws practically contribute to the materialisation of better or worse, and more or less just, health outcomes. This applies to laws that, for example, empower government to implement health protection measures, or to apply taxes to products such as sugary drinks or tobacco to discourage their consumption. It applies to laws that govern interactions of and between private individuals, organisations, corporations, and so on, from public health rationales within torts such as negligence, to areas such as employment law duties. And it applies to criminal law measures around particular forms of harmful behaviours and practices. Secondly, there is law as a constraint to guard against undue interference with individual and commercial freedoms: for example, human rights and equality protections, or wider protections of basic constitutional and common law rights. Notably, within this function too we see the importance of philosophical commitments that are embedded within the idea of law writ large: for example, the rule of law. Thirdly, we have law as a normative system whose study provides its own important measures of critical evaluation in value-based standards of justice. These may come from standards that are internal to (ideas of) law, such as human rights norms or the rule of law. And they also come from critical moral and political theories that help us, as legal scholars, to evaluate, critique, and make practical proposals in relation to law.

Leading scholarship in public health law and legal epidemiology has been particularly attentive to the first and second of these.^125^ It has had important influence in combining questions of understanding from doctrinal legal methods with approaches that look to law as a social phenomenon whose meaning, influence, and practical effect are context-dependent and may change across time. It has also, crucially, combined questions of what law is and does with analyses from (*inter alia*) the health sciences and political sciences to give empirical accounts of laws’ practical effects and influences; and of their *potential*, through litigation, legislation, and distinct methods of implementation. The necessary multi- and transdisciplinarity of studies in law and public health need equally to extend in the directions of philosophy and critical social theory. Works such as the *Lancet*-O’Neill report explicitly note the relevance of the normative questions that would be clustered under our third point in the above list. But much greater attention to and incorporation of such questions within legal scholarship is necessary if we are adequately to frame and respond practically to legal determinants of health injustice.^126^

Many libertarian, and more ‘narrowly liberal’, theories, ideas, and norms, feature in and underpin normative assumptions, both in UK medical law and political morality. This at once perpetuates notions of self-reliance, atomised individualism, and so on, and gives rise to a wariness of the state (or professional hegemonies such as the medical establishment) advancing positions on the ethical values that matter (most), or that act with a paternalistic goal of defining and serving people’s interests. These problems manifest for (potential) laws, in critiques of the idea of law, and as routine assumptions with UK health law scholarship. Nevertheless, we also see sustained, and perhaps growing, concerns about the stark realities of health inequalities, and the structures that contribute to their (worsening) existence. Laws are a fundamental part of the social and institutional architecture within which health inequalities are created and sustained. They may thus also be part of the response to them. Individual laws, law writ large, and the work of legal scholars, have a key part to play in identifying what is meant by health injustice, and how it may be addressed. In this article, we have sought to explain why medical law provides the wrong starting point, what bringing a public health perspective brings to health law scholarship, and outlined four critical, framing questions that must be addressed within such scholarship. The combination of the continued impacts of austerity, the COVID-19 pandemic, the predominance of libertarian ethico-political norms in health policy, and the development and implementation of a new policy agenda underscore the urgency of the project of addressing the legal determinants of health (in)justice.

## Footnotes

1 Commission on Social Determinants of Health, *Closing the Gap in a Generation: Health Equity through Action on the Social Determinants of Health: Commission on Social Determinants of Health Final Report* (WHO 2008).

2 In England, the primary jurisdictional focus of this article, see in particular: Michael Marmot and others, *Fair Society, Healthy Lives: The Marmot Review* (The Marmot Review 2010); Michael Marmot and others, *Health Equity in England: The Marmot Review 10 Years On* (Institute of Health Equity 2020).

3 Norman Daniels, Bruce P. Kennedy and Ichiro Kawachi, ‘Why Justice is Good for Our health: The Social Determinants of Health Inequalities’ (1999) 128(4) Daedalus 215–51.

4 Nancy E Kass, ‘Public Health Ethics: From Foundations and Frameworks to Justice and Global Health’ (2004) 32(2) Journal of Law, Medicine, and Ethics 232–42. Key works at the establishment of the field include: Ronald Bayer and others (eds), *Public Health Ethics: Theory, Policy, and Practice* (OUP 2006); Sudhir Anand, Fabienne Peter and Amartya Sen, *Public Health, Ethics, and Equity* (OUP 2006); Angus Dawson and Marcel Verweij, *Ethics, Prevention, and Public Health* (OUP 2007). This time also marked the publication of the Nuffield Council on Bioethics’ influential report *Public Health—Ethical Issues*, (NCoB, 2007) and the establishment of the journal *Public Health Ethics*. See also John Coggon and Farhang Tahzib, ‘“The Science of Social Justice”: Assuring the Conditions for Ethics and Equity at the Heart of Public Health’ (2021) 43(4) Journal of Public Health e629–e631.

5 John Coggon, *What Makes Health Public?* (CUP 2012).

6 Ruth Faden, Justin Bernstein and Sirine Shebaya, ‘Public Health Ethics’ in Edward N Zalta (ed), *The Stanford Encyclopedia of Philosophy* (Spring 2022 Edition) <https://plato.stanford.edu/archives/spr2022/entries/publichealth-ethics/> accessed 16 November 2022.

7 Sarah Hill, ‘Axes of Health Inequalities and Intersectionality’ in Katherine E Smith, Clare Bambra and Sarah E Hill (eds), *Health Inequalities: Critical Perspectives* (OUP 2015) 95–108. Foundationally on intersectionality in critical legal scholarship, see Kimberlé Crenshaw, ‘Demarginalizing the Intersection of Race and Sex: A Black Feminist Critique of Antidiscrimination Doctrine, Feminist Theory and Antiracist Politics’ (1989) 1 University of Chicago Legal Forum 139–67. See also Beth Wangarĩ Kamunge, *Which Inequalities Should We Focus on in Evaluating Health Policy Before, During, and Following Covid-19?* (UK Pandemic Ethics Accelerator 2021) < https://ukpandemicethics.org/wp-content/uploads/2021/12/Intersectionality-Framework.pdf > accessed 16 November 2022.

8 Townsend Centre for International Poverty Research, ‘Alternative Top Ten Tips for Health’ <www.bristol.ac.uk/poverty/healthinequalities.html> accessed 16 November 2022.

9 See n 4, above.

10 Notable here, amongst others, are the contributions of Lawrence O Gostin and Lindsay Wiley, which focus primarily on US laws and challenge predominant medicalism and individualism, while promoting public health and social justice perspectives: see eg Lawrence O Gostin and Lindsay F Wiley, *Public Health Law: Power, Duty, Restraint* (3rd edn, University of California Press 2016) (the first two editions, published respectively in 2000 and 2008, were single-authored by Gostin, whose foundational work and motivations are well explained in Lawrence O Gostin, ‘From a Civil Libertarian to a Sanitarian’ (2007) 34(4) Journal of Law and Society 594–616); Lindsay F Wiley, ‘Health Law as Social Justice’ (2014) 24(1) Journal of Law and Public Policy 47–105; Lindsay F Wiley, ‘From Patient Rights to Health Justice: Securing the Public’s Interest in Affordable, High-Quality Health Care’ (2016) 37 Cardozo Law Review 833–89. Maxwell Smith has also made significant contributions in exploring the links between philosophical ideas of justice, public health, and policy: see eg Maxwell J Smith, ‘Health Equity in Public Health: Clarifying our Commitment’ (2015) 8(2) Public Health Ethics 173–84. See also the works of Daniel S Goldberg, such as *Public Health Ethics and the Social Determinants of Health* (Springer 2017). Australian scholarship on the links between ethics and social justice, public health, policy, and law similarly boasts important works, such as by Roger Magnusson, including the special issue of Public Health ((2015) 129(8)) that he co-edited with Paul Griffiths on the ‘nanny state’. For a survey of the broad reach of political theories applied to public health, see Coggon, (n 5), ch 8. And reflective of the call to engage works from critical legal theory to analyses of public health and health inequalities, see works such as I Glenn Cohen and others (eds), *Disability, Health, Law and Bioethics* (CUP 2020); Dayna Bowen Matthew, *Just Health: Treating Structural Racism to Health America* (New York University Press 2022).

11 Most notably, see the contributions of Robyn Martin, including her co-edited book Robyn Martin and Linda Johnson (eds), *Law and the Public Dimension of Health* (Cavendish 2001), and, tying this footnote to the previous one, the special issue of Public Health ((2009) 123(3)) co-edited by Belinda Bennett, Lawrence O Gostin, Roger Magnusson, and Robyn Martin. More recently within UK (public) health law scholarship, see eg the political and social justice-oriented analysis of Christopher Newdick, as, eg in his ‘Health Equality, Social Justice and the Poverty of Autonomy’ (2017) 12 Health Economics, Policy and Law 411–33; John Coggon, Keith Syrett and A.M. Viens, *Public Health Law* (Routledge 2017); Michael Thomson, 'Legal Determinants of Health' (2022) Medical Law Review, fwac025, https://doi.org/10.1093/medlaw/fwac025.

12 See nn 10 and 11, as well as contributions regarding legal epidemiology and socio-legal approaches: see especially Scott Burris and others, ‘Making the Case for Laws that Improve Health: A Framework for Public Health Law Research’ (2010) 88(2) Milbank Quarterly 169–210; Scott Burris and others, ‘A Transdisciplinary Approach to Public Health Law: The Emerging Practice of Legal Epidemiology’ (2016) 37 Annual Review of Public Health 135–48; Scott Burris and others, *The New Public Health Law: A Transdisciplinary Approach to Practice and Advocacy* (OUP 2018).

13 Lawrence O Gostin and others, ‘The Legal Determinants of Health: Harnessing the Power of Law for Global Health and Sustainable Development’ (2019) 393 The Lancet 1857–910, 1889.

14 John Coggon, ‘Legal, Moral and Political Determinants within the Social Determinants of Health: Approaching Transdisciplinary Challenges through Intradisciplinary Reflection’ (2020) 13(1) Public Health Ethics 41–47.

15 cf José Miola, *Medical Ethics and Medical Law: A Symbiotic Relationship* (Hart 2007).

16 Department of Health and Social Care, *NHS Constitution for England*, (DHSC: 2012 [last updated January 2021]).

17
*Airedale NHS Trust v Bland* [1993] AC 789 at 796, *per* Sir Stephen Brown P, 833, *per* Hoffmann LJ; 879-80, *per* Browne-Wilkinson L; 896, *per* Mustill L.

18 National Health Act 2006 (as amended), s 1C (emphasis added).

19 Christopher Newdick, *Who Should We Treat?: Rights, Rationing and Resources in the NHS* (2nd edn, OUP 2005); Keith Syrett, *Law, Legitimacy and the Rationing of Health Care: A Contextual and Comparative Perspective* (CUP 2007); Amy Ford, ‘The Concept of Exceptionality: A Legal Farce?’ (2012) 20(3) Medical Law Review 304–36.

20
*Montgomery v Lanarkshire Health Board* [2015] UKSC 11; [2015] AC 1430, *per* Kerr and Reed JJSC, 1459, though see Emily Jackson, ‘Challenging the Comparison in Montgomery between Patients and “Consumers Exercising Choices”’ (2021) 29(4) Medical Law Review 595–612.

21
*Re T (Adult: refusal of medical treatment)* [1992] EWCA Civ 18, [1992] 3 WLR 782, though see John Coggon and Camillia Kong, ‘From Best Interests to Better Interests? Values, Unwisdom and Objectivity in Mental Capacity Law’ (2021) 80(2) Cambridge Law Journal 245–73.

22 The ‘treating patients right’ reference is a (not too oblique) reference to the field-defining works in English medical law of Ian Kennedy: see eg Ian Kennedy, *Treat Me Right: Essays in Medical Law and Ethics* (Clarendon 1991). An irony that bears noting is that while Kennedy’s seminal work has spawned the hard ideological assumptions that we critique in this part of the article, he had also sought (with rather less influence on laws, law, or legal scholarship) to address questions of social injustice and unfair disparities in health given the effects of political and social structures: see Duncan Wilson, *The Making of British Bioethics* (Manchester University Press 2014), chap 3.

23 See Angus Dawson, ‘The Future of Bioethics: Three Dogmas and a Cup of Hemlock’ (2010) 24(5) Bioethics 218–25.

24 John Coggon, ‘Would Responsible Medical Lawyers Lose Their Patients?’ (2012) 20(1) Medical Law Review 130–49.

25 Jonathan Herring, ‘[Review of] *Choosing Life, Choosing Death, The Tyranny of Autonomy in Medical Ethics and Law*, by Charles Foster’ (2010) 30(2) Legal Studies 330–33.

26 See eg Heather Draper and Tom Sorell, ‘Patients’ Responsibilities in Medical Ethics’ (2002) 16(4) Bioethics 335–52; Onora O’Neill, *Autonomy and Trust in Bioethics* (CUP 2002); Margaret Brazier, ‘Do No Harm—Do Patients Have Responsibilities Too?’ (2006) 65(2) CLJ 397–422.

27 Dawson (n 23).

28 Although written against a US context, see Jessica Flanigan, ‘Public Bioethics’ (2013) 6(2) Public Health Ethics 170–84. See also Angus Dawson, ‘Snakes and Ladders: State Interventions and the Place of Liberty in Public Health Policy’ (2016) 42(8) Journal of Medical Ethics 510–13.

29 See Coggon (n 5) chaps 2, and 8–12; Paul Crawshaw, ‘Public Health Policy and the Behavioural Turn: The Case of Social Marketing’ (2013) 33(4) Critical Social Policy 616–37; John Coggon, ‘Smoke Free? Public Health Policy, Coercive Paternalism, and the Ethics of Long-game Regulation’ (2020) 47(1) Journal of Law and Society 121–48.

30 J Adams and others, ‘Why are Some Population Interventions for Diet and Obesity more Equitable and Effective than Others? The Role of Individual Agency?’ (2016) 13(4) PLoS Medicine e1001990.

31 ibid, (references omitted).

32 Department of Health and Social Care, *Working Together to Improve Health and Social Care for All* (DHSC 2021). At the time of writing, it is reported that the direction of policy on this might change away from prioritising promotion of the public’s health: Anon, ‘Anti-obesity Strategy to be Reviewed due to Cost-of-living Crisis’ (2022) *BBC News* <https://www.bbc.co.uk/news/uk-politics-62900076> accessed 16 November 2022.

33 John Coggon, *The Nanny State Debate: A Place Where Words Don’t Do Justice* (Faculty of Public Health 2018).

34 J Bernadette Moore and Charlotte EL Evans, ‘Obese and Hungry: Two Faces of a Nation’ (2020) 370 British Medical Journal m3084.

35 John Coggon, *Government Healthy Weight Strategies: Ethical Considerations* (UK Pandemic Ethics Accelerator 2021) <https://ukpandemicethics.org/wp-content/uploads/2021/05/Government-Healthy-Weight-Strategies.pdf> accessed 16 November 2022.

36 See the papers in the journal special issue on the ‘nanny state’, guest edited by Roger S Magnusson and Paul E Griffiths, of Public Health (2015), 129(8).

37 Generally, see Sridhar Venkatapuram, *Health Justice: An Argument from the Capabilities Approach* (Polity Press 2011).

38 Commission on Social Determinants of Health (n 1).

39 Sridhar Venkatapuram, ‘Global Justice and the Social Determinants of Health’ (2010) 24(2) Ethics and International Affairs 119–30, 127.

40 See above, Section I.

41 Tom L Beauchamp and James F Childress, *Principles of Biomedical Ethics* (8th edn, OUP 2019); within the UK, note especially the advocacy for this approach by Raanan Gillon: see eg Raanon Gillon, ‘Medical Ethics: Four Principles Plus Attention to Scope’ (1994) 309(6948) British Medical Journal 184–88.

42 Robert Beaglehole and others, ‘Public Health in the New Era: Improving Health through Collective Action’ (2004) 363 The Lancet 2084–86; Coggon (n 5), chaps 3 and 8; Alex Mold and others, *Placing the Public in Public Health in Post-War Britain, 1948-2012* (Palgrave MacMillan 2019).

43 Richard Horton, ‘Offline: Apostasy against the Public Health Elites’ (2019) 391 The Lancet 643.

44 Jo Bibby and Sridhar Venkatapuram, *A Recipe for Action: Using Wider Evidence for a Healthier UK: A Collection of Essays Exploring Why we Need Trans-disciplinary Approaches to Improve the Public’s Health* (Health Foundation 2018); Ichiro Kawachi, Iain Lang and Walter Ricciardi, *Oxford Handbook of Public Health Practice* (4th edn, OUP 2020).

45 Gostin and others (n 13).

46 A piece in The Lancet regarding English medical curricula, published in 1907, laments the general understatement given to preventive medicine, and in part of its more detailed comment on educational content states: In so far as the lectures to students are concerned the school committee should endeavour to secure the services of a lecturer who has made a study of the science of preventive medicine rather than of administration and law. The student whose ultimate intention is to embrace general practice has no need or use of the special administrative wisdom of the medical officer of health or a knowledge of the statutes relating to common lodging-houses or the disposal of refuse. Anon, ‘The Teaching of Public Health in Medical Schools,’ The Lancet (1907) 169(4352), 239–240, 239. At the same time (including in the subsequent piece in the issue, regarding the Workmen’s Compensation Act 1906) the journal gave considerable and clear explanatory updates on laws and legal developments.

47 European Convention on Human Rights, art 8. See also arts 9–11.

48 On the reach of public health across distinct points of law and governance, see Coggon, Syrett and Viens (n 11).

49 Beaglehole and others (n 42).

50 See also the review and conceptual analysis of key definitions Marcel Verweij and Angus Dawson, ‘The Meaning of “Public” in “Public Health”’ in A Dawson and M Verweij (eds), *Ethics, Prevention, and Public Health* (OUP 2007).

51 Geoffrey Rose, ‘Sick Individuals and Sick Populations’ (1985) 14(1) International Journal of Epidemiology 32–38.

52 Lawrence O Gostin, ‘Public Health: The “population” as Patient’ in CD DeAngelis (ed), *Patient Care and Professionalism* (OUP 2014).

53 Rose (n 51).

54 Richard Horton, ‘Offline: Where is the Public Health Leadership in England?’ (2011) 378(9796) The Lancet 1060.

55 Hugh Whittall and Dave Archard (on behalf of the members of the Nuffield Council on Bioethics), ‘Statement: COVID-19 and the basics of democratic governance’ *Nuffield Council on Bioethics* (25 April 2020), <https://www.nuffieldbioethics.org/news/statement-covid-19-and-the-basics-of-democratic-governance> accessed 16 November 2022.

56 JP Mackenbach, ‘Politics is Nothing but Medicine at a Larger Scale: Reflections on Public Health’s Biggest Idea’ (2009) 63(3) Journal of Epidemiology and Community Health 181–84. See also Tony Delamothe, ‘Let Us Now Praise Famous Men and Women’ (2012) 345(e7605) British Medical Journal 31.

57 Sally C Davies, and others, ‘For Debate: A New Wave in Public Health Improvement’ (2014) 384(9957) The Lancet 1889–95.

58 Kathryn MacKay, ‘Utility and Justice in Public Health’ (2018) 40(3) Journal of Public Health e413–e418.

59 ibid.

60 John Coggon and AM Viens, *Public Health Ethics in Practice: An overview of public health ethics for the UK Public Health Skills and Knowledge Framework* (Public Health England 2017).

61 Paula Braveman and Sofia Gruskin, ‘Defining Equity in Health’ (2003) 57(4) Journal of Epidemiology and Community Health 254–58.

62 Hill (n 7); Kamunge (n 7).

63 Braveman and Gruskin (n 61).

64 Marmot and others (n 2) 16.

65 Coggon and Tahzib (n 4).

66 Bruce Jennings, ‘Frameworks for Ethics in Public Health’ (2003) 9(2) Acta Bioethica 165–76.

67 Coggon (n 5).

68 See Section II, above.

69 Paula Braveman, Susan Ergerter and David R Williams, ‘The Social Determinants of Health: Coming of Age’ (2011) (32) Annual Review of Public Health 181–98.

70 Sandro Galea and Roger D Vaughan, ‘Causes and Causes of Causes of Population Health: A Public Health of Consequence, March 2018’ (2018) 108(3) American Journal of Public Health 304–05.

71 Academy of Medical Sciences, *Improving the Health of the Public by 2040: Optimising the Research Environment for a Healthier, Fairer Future* (Academy of Medical Sciences 2016).

72 ibid 117.

73 See further Ole Petter Ottersen and others, ‘The Political Origins of Health Inequity: Prospects for Change’ (2014) 383(9917) The Lancet 630–67.

74 Notably, see Department of Health and Social Security, *Inequalities in Health: Report of a Research Working Group*, (DHSS, 1980); Donald Acheson and others, *Independent Inquiry into Inequalities in Health Report* (TSO 1998); Marmot and others (n 2).

75 Public Health England, *Beyond the Data: Understanding the Impact of COVID-19 on BAME Groups* (PHE 2020).

76 Women and Equalities Committee (2020), *Unequal Impact? Coronavirus and BAME People Third Report of Session 2019–21* (HC 2020).

77 Kevin de Sabbata and others, *COVID-19 Policies and their Unequal Impact on the Rights and Dignity of Disabled People* (UK Pandemic Ethics Accelerator 2022) <https://ukpandemicethics.org/wp-content/uploads/2022/07/Disability-project-ethics.pdf> accessed 16 November 2022.

78 Damien Barr, ‘We are in the Same Storm, but not in the Same Boat’ (18 May 2020) <https://eyfs.info/news.html/ eyfsf/we-are-in-the-same-storm-but-not-in-the-sameboat-r295/> accessed 16 November 2022.

79 Zubaida Haque, Laia Becares and Nick Treloar, *Over-Exposed and Under-Protected: The Devastating Impact of COVID-19 on Black and Minority Ethnic Communities in Great Britain* (Runnymede Trust 2020).

80 Carl Baker, and others, ‘COVID-19 and the Digital Divide’ (*POST*, 17th December 2020) <https://post.parliament.uk/covid-19-and-the-digital-divide/> accessed 16 November 2022.

81 See eg Good Law Project, ‘Don’t Let Children go Hungry this Summer’ (9 June 2020) <https://goodlawproject.org/news/children-go-hungry-this-summer/> accessed 16 November 2022.

82 Alan Felstead and others, ‘Unpredictable Times: The Extent, Characteristics and Correlates of Insecure Hours of Work in Britain’ (2020) 51(1–2) Industrial Relations Journal 34–57.

83 Clare Lally, ‘Impact of COVID-19 on Different Ethnic Minority Groups’ (*POST*, 19 October 2020) <https://post.parliament.uk/impact-of-covid-19-on-different-ethnic-minority-groups/> accessed 16 November 2022.

84 Elizabeth J Williamson and others, ‘Factors Associated with COVID-19 Related Death using OpenSAFELY’ (2020) 584 Nature 430–36.

85 Ingrid Torjesen, ‘Covid-19: Middle Aged Women Face Greater Risk of Debilitating Long Term Symptoms’ (2021) 372(829) British Medical Journal.

86 United Nations, *Policy Brief: The Impact of COVID-19 on Women* (9 April 2020) <https://www.un.org/sexualviolenceinconflict/wp-content/uploads/2020/06/report/policy-brief-the-impact-of-covid-19-on-women/policy-brief-the-impact-of-covid-19-on-women-en-1.pdf> accessed 16 November 2022; Mehrunisha Suleman and others, *Unequal Pandemic, Fairer Recovery: The COVID-19 Impact Inquiry Report* (Health Foundation 2021); Office for National Statistics, ‘Coronavirus (COVID-19) and the Different Effects on Men and Women in the UK, March 2020 to February 2021’ (10 March 2021) <www.ons.gov.uk/peoplepopulationandcommunity/healthandsocialcare/conditionsanddiseases/articles/coronaviruscovid19andthedifferenteffectsonmenandwomenintheukmarch2020tofebruary2021/2021-03-10> accessed 16 November 2022.

87 Venkatapuram (n 39).

88 Building in particular on the sorts of approaches listed in the works cited in notes 10–12, above.

89 Patricia H Collins, *Black Feminist Thought: Knowledge, Consciousness, and the Politics of Empowerment* (Routledge 1991) 225.

90 Combahee River Collective, ‘A Black Feminist Statement’ in C Moraga and G Anzaldua (eds), *This Bridge Called My Back: Writings by Radical Women of Color* (Kitchen Table Press 1983 [1977]).

91 Anthony Rafferty, ‘Ethnic Penalties in Graduate Level Over-education, Unemployment and Wages: Evidence from Britain’ (2012) 26(6) Work, Employment and Society 987–1006.

92 Malcolm Brynin and Ayse Güveli, ‘Understanding the Ethnic Pay Gay in Britain’ (2012) 26(4)Work, Employment and Society 574–87.

93 Sharelle Barber, ‘Death by Racism’ (2020) 20(8) The Lancet Infectious Disease 903.

94 Saffron Karlsen, ‘To Address Ethnic Inequalities in COVID-19, we must Acknowledge the Multifaceted Influence of Racism’ (*Bristol Poverty Institute Blog*, 22 April, 2021) <https://bristolpovertyinstitute.blogs.bristol.ac.uk/2021/04/22/to-address-ethnic-inequalities-in-covid-19-we-must-acknowledge-the-multifaceted-influence-of-racism/> accessed 16 November 2022.

95 Dominic Webb, ‘Economic Update: UK Facing “severe recession”’ (UK Parliament, House of Commons Library, 28 May 2020) <https://commonslibrary.parliament.uk/economic-update-uk-facing-severe-recession/> accessed 16 November 2022.

96 David Labord, Will Martin and Rob Vos, ‘Poverty and Food Insecurity could Grow Dramatically as COVID-19 Spreads’ (*International Food Policy Research Institute Blog*, 16 April 2020) <www.ifpri.org/blog/poverty-and-food-insecurity-could-grow-dramatically-covid-19-spreads> accessed 16 November 2022.

97 Commission on Race and Ethnic Disparities, *The Report of the Commission on Race and Ethnic Disparities* (31 March 2021), <https://www.gov.uk/government/publications/the-report-of-the-commission-on-race-and-ethnic-disparities> accessed 16 November 2022.

98 Public Health England, *Disparities in the Risk and Outcomes of COVID-19* (PHE 2020).

99 JS Bamrah and others, ‘The Sewell Report Risks Turning the Clock Back on the Fight against Racism in the UK’ (*BMJOpinion*, 12 April 2021) <https://blogs.bmj.com/bmj/2021/04/12/the-sewell-report-risks-turning-the-clock-back-on-the-fight-against-racism-in-the-uk/> accessed 16 November 2022.

100 National Audit Office, *Readying the NHS and Adult Social Care in England for COVID-19* (NAO 2020).

101 House of Commons Health and Social Care, and Science and Technology Committees, *Coronavirus: Lessons Learned to Date* (HC92 2021) 6.

102 This is not to suggest that some poor people are more ‘deserving’ than others of state support.

103 Hannah Lambie-Mumford, ‘The Growth of Food Banks in Britain and What they Mean for Social Policy’ (2019) 39(1) Critical Social Policy 3–22.

104 Beth W Kamunge, ‘*Written submission in response to the ‘An Equal Recovery’ Parliamentary Consultation by Treasury Committee*,’ (2021), report submission number AER0039 <https://committees.parliament.uk/writtenevidence/37923/pdf/> accessed 16 November 2022.

105 Premila Webster and Keith Neal, ‘“Normal led to this”-Where Next?’ (2021) 43(2) Journal of Public Health 217–18.

106 Charles W Mills, *The Racial Contract* (Cornell University Press 1997).

107 See n 10, above.

108 See n 11, above.

109 Kamunge (n 7).

110 Suleman and others (n 86).

111 Anon, ‘George Floyd Death: Thousands Turn out for UK Anti-racism Protests’ (*BBC News*, 7 June 2020) <www.bbc.co.uk/news/uk-52949014> accessed 16 November 2022.

112 Martin McKee and David Stuckler, ‘Revisiting the Corporate and Commercial Determinants of Health’ (2018) 108(9) American Journal of Public Health 1167–70.

113 cf Devyani Prabhat, ‘Unequal Citizenship and Subjecthood: A Rose by any Other Name…?’ (2020) 71(2) Northern Ireland Legal Quarterly 175–91.

114 Gayatri Chakravorty Spivak, ‘Can the Subaltern Speak?’ (1988) 14(27) Die Philosophin 42–58.

115 Kristie Dotson, ‘Conceptualising Epistemic Oppression’ (2014) 28(2) Social Epistemology 115–38.

116 Miranda Fricker, *Epistemic Injustice: Power and the Ethics of Knowing* (OUP 2007).

117 Karlsen (n 94).

118 Dorothy Roberts, *Fatal Invention* (New Press 2012).

119 Nuffield Council on Bioethics (n 4), ch 2; Coggon (n 5), ch 8.

120 See nn 10–11 above.

121 Gostin and others (n 13).

122 See eg the sources cited at <https://www.parliament.uk/business/publications/coronavirus/inquiries-and-reports/> accessed 16 November 2022.

123 See eg the resources generated by the UK Health Foundation collected here: <https://www.health.org.uk/what-we-do/responding-to-covid-19> accessed 16 November 2022.

124
*R (Dolan and others) v Secretary of State for Health and Social Care and another* [2020] EWCA Civ 1605; [2021] 1 WLR 2326.

125 Burris and others (n 12); Gostin and others (n 13).

126 Coggon (n 14).

